# The Human Microbiome Project: A Community Resource for the Healthy Human Microbiome

**DOI:** 10.1371/journal.pbio.1001377

**Published:** 2012-08-14

**Authors:** Dirk Gevers, Rob Knight, Joseph F. Petrosino, Katherine Huang, Amy L. McGuire, Bruce W. Birren, Karen E. Nelson, Owen White, Barbara A. Methé, Curtis Huttenhower

**Affiliations:** 1 The Broad Institute of MIT and Harvard, Cambridge, Massachusetts, United States of America; 2 Department of Chemistry and Biochemistry, University of Colorado, Boulder, Colorado, United States of America; 3 Howard Hughes Medical Institute, Boulder, Colorado, United States of America; 4 Human Genome Sequencing Center, Baylor College of Medicine, Houston, Texas, United States of America; 5 Molecular Virology and Microbiology, Baylor College of Medicine, Houston, Texas, United States of America; 6 Alkek Center for Metagenomics and Microbiome Research, Baylor College of Medicine, Houston, Texas, United States of America; 7 Center for Medical Ethics and Health Policy, Baylor College of Medicine, Houston, Texas, United States of America; 8 J. Craig Venter Institute, Rockville, Maryland, United States of America; 9 Institute for Genome Sciences, University of Maryland School of Medicine, Baltimore, Maryland, United States of America; 10 Biostatistics, Harvard School of Public Health, Boston, Massachusetts, United States of America

## Abstract

This manuscript describes the NIH Human Microbiome Project, including a brief review of human microbiome research, a history of the project, and a comprehensive overview of the consortium's recent collection of publications analyzing the human microbiome.

The Human Microbiome Project (HMP) [1],[2] is a concept that was long in the making. After the Human Genome Project, interest grew in sequencing the “other genome" of microbes carried in and on the human body [3],[4]. Microbial ecologists, realizing that >99% of environmental microbes could not be easily cultured, developed approaches to study microorganisms in situ [5], primarily by sequencing the 16S ribosomal RNA gene (16S) as a phylogenetic and taxonomic marker to identify members of microbial communities [6]. The need to develop corresponding new methods for culture-independent studies [7],[8] in turn precipitated a sea change in the study of microbes and human health, inspiring the new term “metagenomics" [9] both to describe a technological approach—sequencing and analysis of the genes from whole communities rather than from individual genomes—and to emphasize that microbes function within communities rather than as individual species. This shift from a focus on individual organisms to microbial interactions [10] culminated in a National Academy of Science report [11], which outlined challenges and promises for metagenomics as a way of understanding the foundational role of microbial communities both in the environment and in human health.

Pioneering medical microbiologists applied these approaches, finding far more microbial diversity than expected even in well-studied body site habitats [12]. Technological advances further enabled sequencing of communities across the human body, and immunologists began exploring the fundamental role of microorganisms in the maturation of the innate and adaptive immune systems. Initial metagenomic studies of human-associated microbial communities were performed using the traditional Sanger platform [13],[14]. Upon introduction of pyrosequencing [15], the number of 16S-based data sets increased dramatically [16],[17]. The time was right to invest in a concerted study of the microbial communities associated with the human body and the metabolic capabilities they provide—the human microbiome (Figure 1) [18].

**Figure 1:**
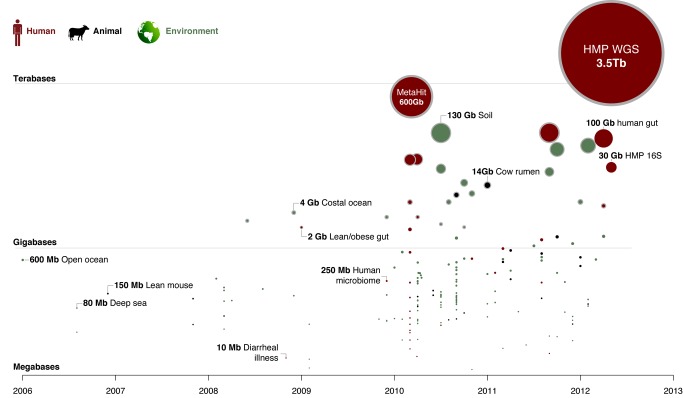
Timeline of microbial community studies using high-throughput sequencing. Each circle represents a high-throughput sequence-based 16S or shotgun metagenomic bioproject in NCBI (May 2012), indicating the amount of sequence data produced for each project (circle area and *y*-coordinate) at the time of publication/registration (*x*-coordinate). Projects are grouped by human-associated (red), other animal (black), or environmental (green) communities, and shotgun metagenomic projects are marked with a grey band. Selected representative projects are labeled: open ocean [68], deep sea [69], lean mouse [70], diarrheal illness [71], costal ocean [72], lean/obese gut [53], human microbiome [56], MetaHIT (gut) [58], cow rumen [73], soil (NCBI BioProject PRJNA50473), and human gut [74]. Note that HMP has deposited a total of 7.44 terabases of shotgun data in SRA, of which 49% is host DNA derived data that was filtered and only available through protected access in dbGaP project phs000228.

To coordinate these efforts relating the microbiome to human health, the NIH Common Fund launched the HMP as a community resource program (http://commonfund.nih.gov/hmp/) [19]. One of its main goals was to create a baseline view of the healthy human microbiome in five major areas (airways, skin, oral cavity, gastrointestinal tract, and vagina) and to make this resource available to the broad scientific community. Characterizing the baseline state of the microbiota is a critical first step in determining how altered microbial states contribute to disease (e.g., [13],[20]–[23]). Previous work showed wide inter- and intra-personal diversity of human-associated microbes [24], necessitating analysis of a large number of subjects and characterization of many reference bacterial genomes [25] to assist in interpretation of metagenomic data. The scope of the HMP thus required a particularly diverse consortium (Figure 2A), and collaboration among these teams ultimately stimulated research growth throughout the field and produced a study including the first consistent sampling of many clinically relevant body habitats, within a large population, with paired 16S profiling and deep metagenomic sequencing coverage for hundreds of microbial communities.

**Figure 2:**
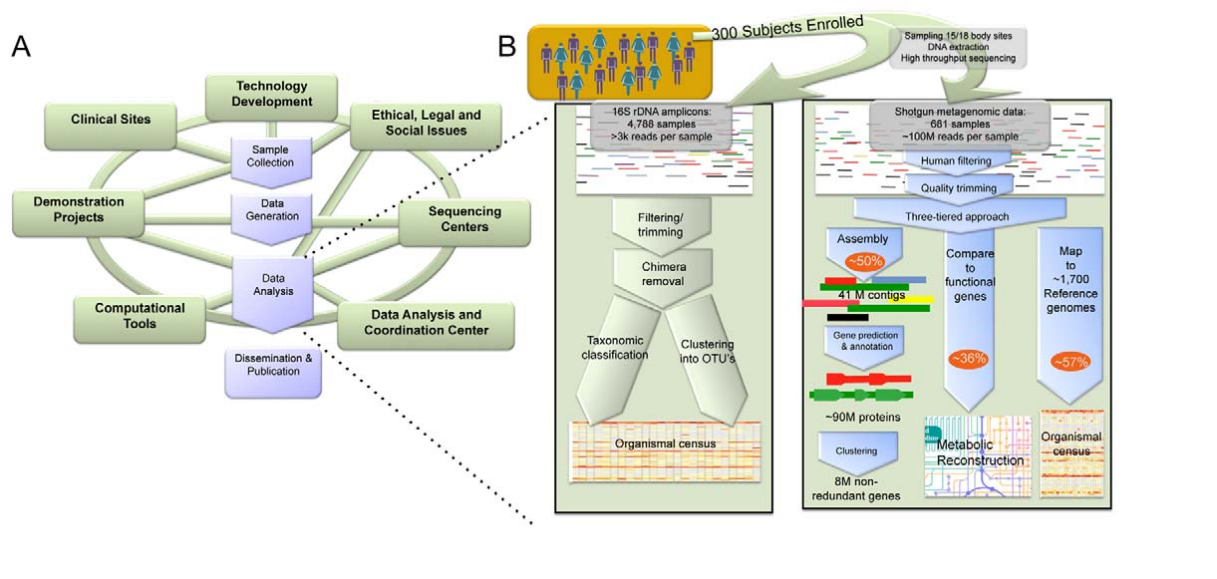
HMP consortium activities as a model for microbiome data generation and analyses. (A) Initiatives within the HMP coordinated to isolate samples, generate data, perform analysis, and publish results. *Technology development* was employed to develop novel bacterial culture and DNA isolation techniques. *Ethical Legal and Social Implications (ELSI)* work anticipated societal implications and guided policies associated with human subject microbiomes. *Clinical sites* were collected samples from large cohorts of healthy individuals, with nucleotide sequence information derived at four *sequencing centers* at the Baylor College of Medicine (BCM), the Broad Institute, the J. Craig Venter Institute (JCVI), and the Washington University Genome Institute (WUGI). Additional *demonstration projects* assessed primarily microbiome alterations related to disease. In addition to analysis throughout the HMP consortium, *computational tools* were funded to address, for example, genome assembly, microbial ecology, and statistical modeling. A *data analysis and coordination center* provided a portal to all data generated. (B) Overview of the analysis approaches that were the ultimate product of the HMP consortium, corresponding to data products and protocols available at http://hmpdacc.org.

The HMP required careful consideration of ethical, legal, and social implications (ELSI) unique to the study of the microbiome [26]. Such research raises questions regarding traditional distinctions between self and non-self, human and non-human, genetics and environment, and health and disease. The prospect of manipulating the microbiota in ways that could permanently alter an individual's biological identity requires the development of new ethical paradigms analogous to, but not identical to, those already considered for gene therapy. Likewise, just as gene patents have proven controversial, defining who “owns" a microbiome raises difficult questions of intellectual property. The ELSI team helped to develop an appropriate sample collection protocol, to draft a template for informed consent, and consulted on ethical issues arising during the study, such as the possibility that unique human microbiome “signatures" [27] might compromise participant privacy. A portion of the HMP's dedicated research budget continues to be committed to integrating multidisciplinary approaches (including philosophical, social science, and legal methods) to study these issues and involve stakeholders including study participants, scientists, policy makers, patients, and indigenous populations.

## Planning for Human Microbiome Studies: Tools, Techniques, and Design

Any study of human populations must put both subject protection and study design first, and the HMP was no exception. Power calculations for microbiome studies in human cohorts are particularly challenging, as they must simultaneously address assay types (e.g., 16S versus shotgun), depth of sequencing, taxon detection, and fold abundance changes in clades, genes, or pathways of interest [28]–[31]. After study design, as the HMP spanned multiple sequencing centers over a prolonged duration, the group established standardized and benchmarked protocols for sample collection [2], handling, and subsequent 16S profiling [32]. Metagenomic library construction was likewise standardized among centers, and stringent quality control was aided by the optimization of 16S read processing [33] and by improved taxonomic frameworks for classification of microbial sequences prior to biological interpretation [34].

Finally, quality data generation from appropriately designed microbiome studies enables a variety of subsequent computational analyses (Figure 2B). While we refer the reader to existing broader reviews of human microbiome bioinformatics [35]–[37], here we highlight numerous recent approaches specifically developed during the HMP. Several of these focused on microbial interactions, such as ecological network reconstruction [38],[39]. Other computational methods dealt with metagenomic sequences, including both assembly-based [40],[41] and assembly-free analyses of microbial community membership [42] and metabolic function [43]. Both data types enable taxonomic and phylogenetic profiling [44],[45], and ecological metrics proved to associate microbial, gene, and pathway diversity on an unprecedented scale [2]. The HMP Data Analysis Coordination Center (DACC, http://hmpdacc.org) hosts all available HMP data and many tools, focusing the tremendous quantity of raw data through lenses such as SitePainter [46]; IMG/HMP, an HMP-specific version of the Integrated Microbial Genomes (IMG [47]) system; METAREP [48]; and MG-RAST [49], and efforts are ongoing to provide these data for meta-analysis alongside other human microbiome studies in the cloud.

## Community Structure, Function, and a “Core" Human Microbiome

The HMP was designed in part to address a key question about our microbial selves: do all humans have an identifiable “core" microbiome of shared components comparable to our shared genome [50]? Several definitions of “core" have been proposed, recently unified in one conceptual framework [51]. Earlier studies reported that different people shared few microbes in their gut and skin microbiota [17],[52]–[56], a greater fraction of their oral microbiota [56],[57], or might be classifiable into multiple core microbiomes based on vaginal [20] and gut communities [58]. The HMP provides a comprehensive picture of the human microbiome covering multiple body sites and thus an in-depth exploration of these concepts. The study confirmed high inter-individual variation [59] and showed that even rare organisms in these communities are important reservoirs of genetic diversity [60]. Additionally, the large HMP cohort shows that the composition of the gut microbiome rarely clusters subjects into discrete types, as was suggested before on more limited data [61]; although other habitats such as the vagina can exhibit such clustering [20], the gut was most often characterized by smooth abundance gradients of key organisms [2].

A potentially more universal “core" human microbiome emerged during the consideration of microbial genes and pathways carried throughout communities' metagenomes. While microbial organisms varied among subjects as described above, metabolic pathways necessary for human-associated microbial life were consistently present, forming a functional “core" to the microbiome at all body sites [2],[43],[53]. Although the pathways and processes of this core were consistent, the particular genes that implemented them again varied. Microbial sugar utilization, for example, was enriched for metabolism of simple sugars in the oral cavity, complex carbohydrates in the gut, and glycogen/peptidoglycan degradation in the vaginal microbiome [62]. The healthy microbiome may thus achieve a consistent balance of function and metabolism that is maintained in health, but with fine-grained details personalized by genetics, early life events, environmental factors such as diet, and a lifetime of pharmaceutical and immunological exposures [41].

## The Healthy Microbiome Informs Studies of Disease

Data from individuals without overt signs of disease serve as an excellent reference for disease-associated microbiome studies, while also providing a comprehensive baseline for comparison of Western populations with disparate geographic, ethnic, and genetic cohorts [63]. The adoption of uniform sampling, nucleic acid extraction, sequencing, and analysis protocols is an important step in such integration, with some success already realized in, for example, several aspects of autoimmune disease. The inflammatory bowel diseases have long been linked to the human gut microbiome [22], with integration of host genotype, gene expression, and microbial membership now suggesting mediation of specific host-microbial interactions by human gene products as well as by host environment [64],[65]. Bacteria are of course not the only mediators of dysbiotic disease, and metagenomic approaches can also be used to identify potential viral etiologies (e.g., in pediatric fever of undefined origin [66]). Likewise the “healthy" microbiome provides a baseline not only for integration with disease-related studies, but for broader populations such as a recent comparison using HMP protocols among a cohort of pregnant women [67]. The normal variation of the microbiome within healthy states and its potential misregulation in disease is thus being pursued in earnest, as related laboratory and computational methods continue to be adapted to better characterize the impact of bacteria, archaea, viruses, and fungi throughout human body habitats.

The HMP has thus greatly advanced our knowledge of the microbes in a healthy adult reference population, and provided much-needed infrastructure in terms of reference genomes, laboratory protocols, computational methods, and ELSI considerations [1],[2] to help enable a vast range of studies that will likely find associations between human-associated microbial communities and disease. The next steps will be to discover which of these microbial community changes result from disease and which cause it, to understand how healthy variation relates to variation within the context of different disorders, and to use a combination of laboratory and computational techniques to begin unraveling causal mechanisms on levels ranging from the molecular to the societal. In particular, the study of individuals of all ages and across cultures, together with prospective longitudinal studies and careful work in in vitro and animal models, will be critical to developing both the science and the technology that will allow us to alter our microbial genomes, far easier to alter than the host genome within each of our “human" cells, in order to maintain and improve health.

## Abbreviations

16S: 16S ribosomal RNA gene
DACC: HMP Data Analysis Coordination Center
ELSI: Ethical Legal and Social Implications
HMP: Human Microbiome Project
